# Role modelling to support careers in general practice: a realist review protocol

**DOI:** 10.3399/BJGPO.2024.0109

**Published:** 2024-10-02

**Authors:** Elizabeth Iris Lamb, Bryan Burford, Catherine Exley, Gillian Vance, Valerie Wass, Hugh Alberti

**Affiliations:** 1 School of Medicine, Faculty of Medical Sciences, Newcastle University, Newcastle upon Tyne, UK; 2 Population Health Sciences Institute, Faculty of Medical Sciences, Newcastle University, Newcastle upon Tyne, UK; 3 School of Medicine, Keele University, Keele, UK

**Keywords:** undergraduate education, education, family medicine, general practice, students, medical, systematic reviews

## Abstract

**Background:**

Role models encountered during undergraduate training play an important part in shaping future doctors. They can act as powerful attractants towards, and deterrents away from, a career in general practice. Many GP educators, who act as role models, are burnt-out and wish to leave the profession, which may limit their ability to influence students positively, with consequent detrimental impact on recruitment to the specialty.

**Aim:**

A realist review will be undertaken, aiming to explore how, why, and for whom role modelling in undergraduate medical education can support medical students towards careers in general practice.

**Design & setting:**

The realist review will follow Pawson’s five steps, including: locating existing theories; searching for evidence; article selection; data extraction; and synthesising evidence and drawing conclusions. It will explore literature published in the English language between 2013 and 2024.

**Method:**

An initial explanatory framework (initial programme theory; IPT) will be developed, guided by a stakeholder panel including medical undergraduates, GPs, and patient and public representatives. Searches will be developed and conducted in electronic databases and grey literature. Studies will be included if they explore the relationship between GP role modelling and undergraduate career choice, and relevant data will be extracted.

**Conclusion:**

Findings will refine the IPT, unveiling key contexts, mechanisms, and outcomes that influence role modelling in undergraduate GP medical education and support or deter students from careers in general practice. These findings will support recommendations and interventions to facilitate positive outcomes, including improved recruitment to general practice.

## How this fits in

Role modelling has a powerful impact on the career decisions of medical undergraduates.^1^ It is important that medical students are supported towards careers in general practice in order to ensure there are adequate numbers of GPs to meet the UK population's healthcare needs.^2^ This research will explore how, why, for whom, and to what extent role modelling in undergraduate medical education can be maximised to support careers in general practice. This will have relevance to educators, policymakers, and all GPs involved in teaching medical students.

## Introduction

Recruiting more GPs to the workforce has been a key priority for the UK government and internationally, yet strategies have not translated into significantly increased numbers, with growth of this area of the workforce in the UK stagnating for many years.^3^ Targets state that 50% of doctors should enter training to become a GP,^2^ yet actual numbers are closer to 30%.^4^ Decisions on choice of medical specialty are usually made as undergraduates and 65% of graduates do not then change their specialty preference,^5^ therefore intervening in undergraduate training has potential to support future doctors towards careers in general practice.

Role modelling has been identified as a crucial yet often hidden aspect of undergraduate training, with great potential to encourage medical students to choose a career in general practice,^1,6^ in addition to early and significant exposure to the specialty during training.^7^ Unfortunately, negative encounters with GP role models can deter undergraduates from the career.^8^ Data from the UK regulator, the General Medical Council (GMC), suggest that more than 40% of those who leave the GP workforce do so owing to burnout.^9^ With the highest levels of burnout of all medical specialties,^10^ there is considerable risk that undergraduates encounter burnt-out GPs on placements who may struggle to act as positive role models.

The importance of role modelling is emphasised in the 2016 Medical Schools Council (MSC) and Health Education England (HEE) report, *By choice, not by chance,* which made a series of recommendations to support medical students towards careers in general practice.^11^ These included the following: increasing the visibility of positive and enthusiastic GP role models in educational institutions; raising the profile of academic role models; and tackling denigration of the specialty. UK medical schools have taken a variety of actions to implement the recommendations.^12^ This realist review will build on existing evidence to explore how, why, and for whom role modelling in undergraduate medical education can support (or deter) medical students towards careers in general practice. Findings will support the development of recommendations and interventions to maximise the potential of positive role modelling to support careers in general practice.

The research questions are as follows:

1. What are the potential outcomes of GP role modelling in undergraduate medical education?
2. What are the key contexts where GP role modelling occurs to produce intended and unintended outcomes?
3. What are the key mechanisms whereby GP role modelling leads to intended and unintended outcomes?
4. In what ways can interventions support positive GP role modelling to facilitate careers in general practice and reduce unintended outcomes?

## Method

Realism recognises that interventions are not universally successful and work better in some circumstances than in others.^13^ A realist approach is ideal for exploring a complex intervention, such as role modelling in medical education, as it helps to understand what works, for whom, in what circumstances, and why.^14^ A realist review of existing literature will facilitate exploration of potential contexts, mechanisms, and outcomes contributing to the intended and unintended outcomes of GP role modelling in undergraduate medical education. The review will follow Pawson’s five steps for realist reviews:^15^ locating existing theories; searching for evidence; article selection; data extraction; and synthesising evidence and drawing conclusions. The review will adhere to the quality standards for realist synthesis outlined in the RAMESES (Realist And Meta-narrative Evidence Syntheses: Evolving Standards) project.^16^

### Stakeholder and patient–public input

**Figure 1.**
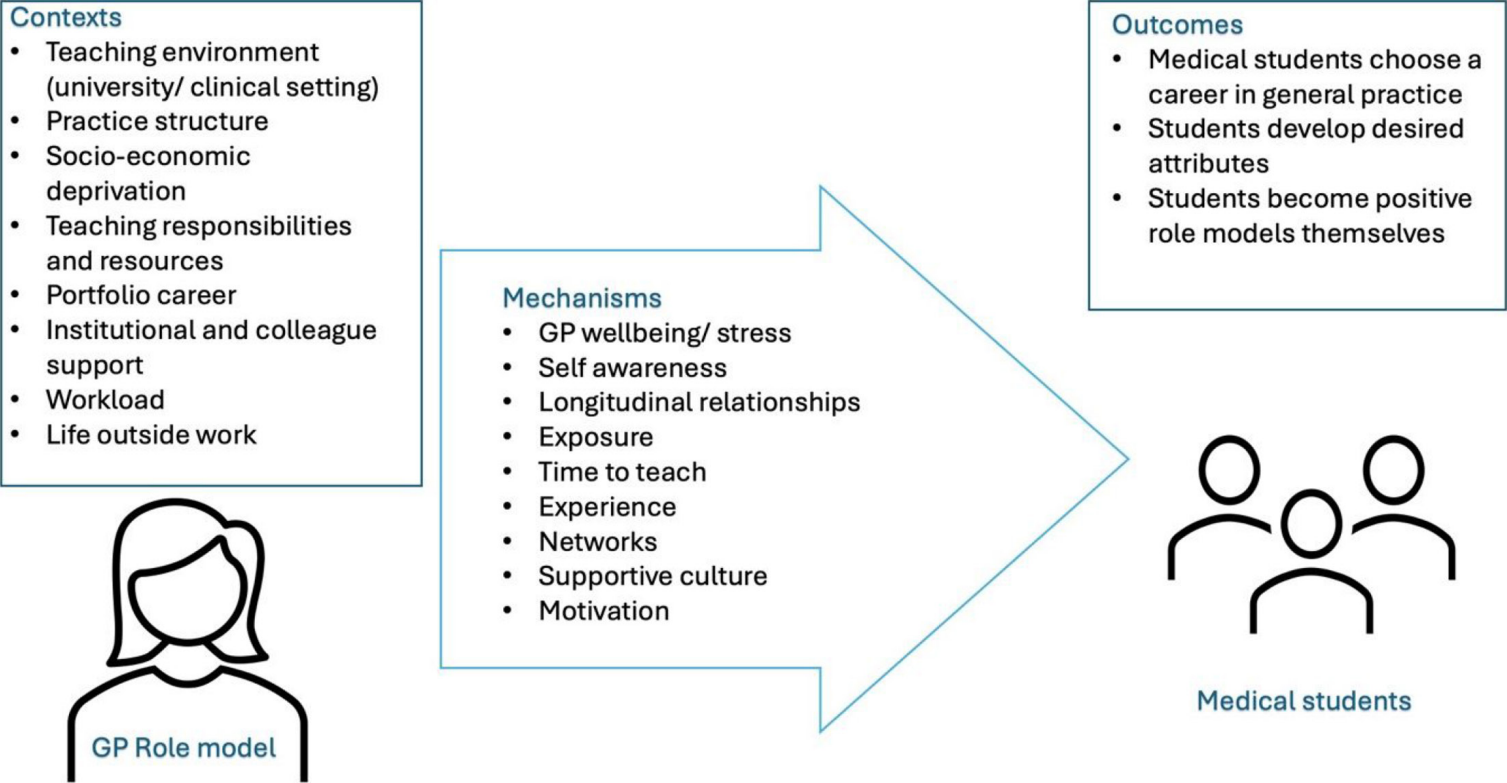
Initial programme theory demonstrating potential contexts, mechanisms and outcomes for exploration in this realist review

Integrated knowledge transition principles will be applied^17^ to facilitate co-produced research where stakeholders are involved in all parts of the research process, with the aim of increasing the impact of the work. A group of stakeholders and patient and public involvement (PPI) participants has been formed in the preparation stages of this project and will contribute to the generation of knowledge^18^ through three meetings during the review process. The PPI group comprises five members of the public who all have experience of using general practice services; the group has been selected to represent a diverse population, including representatives from areas of socioeconomic deprivation. The stakeholder group has been selected to represent those with a stake in the education of the future GP workforce, including those who have actively chosen not to become a GP. The group consists of a GP working in an area of socioeconomic deprivation, a GP trainee, four medical students from different northern medical schools, a senior GP leader within an undergraduate institution, a secondary care representative, a practice manager, and a senior GP undergraduate teacher. Initial meetings with the stakeholder and PPI group have contributed to the initial programme theory (IPT) (Figure 1) and developing the search strategy. Both groups will support with developing context–mechanism–outcome configurations (CMOCs) as the review progresses and with translating findings into recommendations and potential interventions.

### Setting

The review will explore literature published between 2013 and 2024 in the English language and will include grey literature. This period has been selected as in 2013 the *Shape of training* review was published, a key report that impacted on the medical education landscape, highlighting the importance of education in *‘ensuring doctors are trained with appropriate skills, competencies and aptitudes to meet changing needs*’.^19^

While literature describing UK-based undergraduate education will be the main focus, a worldwide search will be undertaken, as there may be areas of relevance to the developing programme theory, particularly in descriptions of interventions to support role modelling.

### Participants

The populations to be considered are GPs and undergraduate medical students; the intervention is role modelling; and the outcomes will include, but are not limited to, career choice. The search strategy is to be undertaken in MEDLINE initially and replicated in other databases, and is described in Table 1.

**Table 1.** Realist search strategy

MEDLINE search term(s)	
1	exp Education, Medical, Undergraduate/
2	exp Students, Medical/
3	exp Schools, Medical/
4	medical student.mp.
5	1 or 2 or 3 or 4
6	exp general practice/ or exp family practice/
7	general practice.mp.
8	exp Family Practice/ or exp Physicians, Family/ or exp Primary Health Care/
9	6 or 7 or 8
10	family practice.mp.
11	family medicine.mp.
12	10 or 11
13	9 or 12
14	mentor.mp. or exp Mentors/
15	role model.mp.
16	role model*.mp.
17	supervisor.mp.
18	teacher.mp.
19	faculty, medical/ or health educators/
20	14 or 15 or 16 or 17 or 18 or 19
21	5 and 13 and 20
22	limit 21 to (english language and yr="2013 -Current")

### Information sources

The following electronic databases will be searched: MEDLINE, Embase, CINAHL, ERIC (Education Resources Information Center), the Cochrane Library, and APA PsycInfo. Overton electronic database will be used to identify grey literature, and online magazines *GP Online* and *Pulse* will be hand-searched. Policy documents will be identified through searching websites including The King’s Fund, General Medical Council, and Medical Schools Council. The AI search engine Consensus (version 2.0) will also be used for identifying further literature of relevance. Reference lists of included articles will be screened for further articles of relevance.

### Article selection

Table 2 shows the inclusion and exclusion criteria that have been developed by the research team, with stakeholder and PPI input, based on the IPT.

**Table 2.** Article inclusion and exclusion criteria

Inclusion:
Published between 2013 and 2024 AND
Focus on undergraduate training delivered by GPs AND
Includes information on how GPs may be seen by medical students (which may or may not be explicitly described as role modelling) OR
Includes information on interventions to support GPs in their educational role OR
Includes information linking general practice career choice (towards or against) with GPs encountered during undergraduate training OR
Includes information linking other key outcomes (professional identity formation, development of professional attributes and so on) with undergraduate training delivered by GPs

**Exclusion:** Not published in English language
Focus on postgraduate rather than undergraduate training
Describing training of non-medical undergraduates
Describes training of undergraduates in general practice by educators who are not GPs
No focus on medical education
No focus on role modelling or similar student–educator relationship

### Data management

Data will be managed using Rayyan software. Search results will be imported to EndNote 21, then exported into Rayyan once all searches are completed. De-duplication will take place, followed by screening of titles and abstracts.

### Selection process

Using the inclusion and exclusion criteria outlined in Table 2, articles will be excluded, included, or assigned to a ‘maybe’ category by the lead author. Those in the included and maybe categories will then be screened at full text to make a final decision on inclusion or exclusion. Other authors will participate in article screening, with a 5% sample shared between them for secondary screening at both title and abstract and full-text screening. Rayyan software will support the allocation of this sample and will blind the secondary screeners to the primary screener decision. Where there are discrepancies in decisions, these will be discussed among the whole team. Articles will be selected for inclusion in the realist review following an assessment of each document’s relevance (whether it contains data relating to relevant contexts, mechanisms, or outcomes or the relationships between these) and rigour (whether the methods used to generate each piece of data are credible and trustworthy).^15^ A wide range of documents will contribute to the realist review, which may include primary research studies, policy documents, news articles, websites, and opinion pieces.

### Data extraction

Data will be extracted into a data-extraction form, which will be refined and piloted before use. Data will be extracted by the lead author, with a further 5% sample reviewed independently by other authors. Extracted data may consist of descriptions of role modelling interventions, outcomes of interventions, or explanations about how and why role modelling interventions have worked in particular contexts.^20^ Data that support the use of realist logic to answer the review questions will be of particular interest; for example, data on CMOCs, demi-regularities (semi-predictable patterns of outcomes), and programme theories.^20^ If articles are unavailable online, authors will be contacted directly. The realist review process is iterative and as the programme theory develops, data will be collected to test the emerging programme theory and reveal underlying mechanisms, therefore the data extracted are likely to change as the review progresses.

### Data synthesis

Data synthesis will aim to use collected data to further develop and refine the IPT. Through iterative data collection and synthesis, the IPT will be developed into a series of CMOCs, which will then be tested through further data collection to confirm or refute the CMOCs. Included articles will be considered for data that may be interpreted as functioning as a context or outcome, and then interpretations will be made on how these contexts and outcomes link via a mechanism. Judgements will then be made about how CMOCs relate to the IPT. During analysis, codes will be developed through inductive (data from documents), deductive (from the programme theory) and retroductive (interpretations of the data on underlying mechanisms causing contexts to lead to outcomes) reasoning. Codes will be refined as concepts emerge throughout the analysis, and quotes from the literature assigned to the codes as supporting evidence.

The research team, and the stakeholder and PPI groups will all be involved in the process of data synthesis, development of CMOCs, and causal pathways, bringing their experience to the decision-making process and resolving discrepancies through discussion. Comparisons will be made across the pool of literature to understand how and why observed outcomes occur. For example, we will compare documents that describe different delivery methods of teaching in general practice or types of general practice placement, to understand how contexts affect GP educator role modelling and subsequently influence on undergraduate career choice. Findings will be assimilated as a refined programme theory, which will then be tested with a subsequent realist evaluation.

## Discussion

This realist review will build on existing literature, seeking to understand how, why, and for whom role modelling in undergraduate medical education can support medical students towards careers in general practice. Understanding the key contexts, mechanisms, and outcomes in the existing evidence base will support the development of recommendations and interventions to maximise the potential of this powerful and complex educational intervention. It will be of interest to educators and policymakers who are interested in securing the future GP workforce.

### Strengths and limitations

This is the first realist review to be undertaken and published in this area. A realist approach is a methodological strength as it will seek to look beyond *what* works, to explore *how*, *why*, and *for whom* it works, and to inform educational policy as a result.

The fact this review is being conducted at a time of rapid change in undergraduate medical education may be both a strength and a limitation. Included literature may become less relevant as educational models change, but the iterative realist approach should support focus on specific areas of the programme theory as their relevance becomes more apparent.

Time and funding constraints mean that it will not be possible to explore literature not published in English. However, the searches will allow international literature to be included, as there is likely to be learning in the medical educational approaches taken by other countries.

## Trial registration number

This review protocol is published in the International Database of Education Systematic Reviews, IDESR00125.

## Data

The dataset relied on in this article is available from the corresponding author on reasonable request.
